# Titano-magnetite nanoparticles for supercapacitors and magnetic hyperthermia performance

**DOI:** 10.1038/s41598-025-08082-3

**Published:** 2025-07-02

**Authors:** Amol B. Pandhare, Swapnajit V. Mulik, Prashant N. Nikam, Satish S. Phalake, Vishwajeet. M. Khot, Manikandan Ayyar, Senthilkumar Ramasamy, Sagar D. Delekar, Rajendra P. Patil, P. Prabhu, Abdullah M. S. Alhuthali, Magda H. Abdellattif, M. Khalid Hossain

**Affiliations:** 1 Department of Chemistry, Shivaji University, Kolhapur, MS 416 004 India; 2 Department of Chemistry, M. H. Shinde Mahavidyalaya, Tisangi, Gaganbavda, Kolhapur, MS 416 206 India; 3 Dattajirao Kadam Arts, Science and Commerce College, Ichalkaranji, MS 416115 India; 4 Department of Physics, Shivaji University, Kolhapur, MS 416 004 India; 5 Center for Interdisciplinary Research, D.Y. Patil Education Society Deemed University, Kolhapur, MS 416 006 India; 6 Department of Chemistry, Centre for Material Chemistry, Karpagam Academy of Higher Education, Coimbatore, Tamil Nadu 641 021 India; 7 Center of Excellence in Advanced Materials and Green Technologies, Amrita School of Engineering, Amrita Vishwa Vidyapeetham (Coimbatore Campus), Amrita Vishwa Vidyapeetham, Coimbatore, Tamil Nadu 641 112 India; 8 Research and Innovation Cell, Rayat Bahra University, Mohali-140301, Punjab India; 9 Department of Mechanical Engineering, Mattu University, 318 Mettu, Ethiopia; 10 Department of Physics, College of Sciences, Taif University, P.O. Box 11099, 21944 Taif, Saudi Arabia; 11 Department of Chemistry, College of Sciences, University College of Taraba, Taif University, P.O. Box 11099, 21944 Taif, Saudi Arabia; 12 Institute of Electronics, Atomic Energy Research Establishment, Bangladesh Atomic Energy Commission, Dhaka, 1349 Bangladesh; 13 Department of Advanced Energy Engineering Science, Interdisciplinary Graduate School of Engineering Sciences, Kyushu University, Fukuoka, 816-8580 Japan

**Keywords:** Biosynthesis, Supercapacitor, Ti_0.5_Fe_2.5_O_4_ NPs, Magnetic hyperthermia, Specific loss power, Chemistry, Materials science, Physics

## Abstract

This study investigates the dual functionality of titano-magnetite (Ti_0.5_Fe_2.5_O_4_) nanoparticles (NPs) synthesized via a green approach for both magnetic hyperthermia and energy storage applications. Spherical Ti_0.5_Fe_2.5_O_4_ NPs with an average crystallite size of 10–15 nm were successfully synthesized using pomegranate fruit shell extract as a natural reducing and capping agent. The synthesized NPs exhibited superparamagnetic behavior, with a saturation magnetization value of 32.2 emu/g at 300 K. When exposed to an alternating current (AC) magnetic field of 20–26.67  kA/m at 277 kHz, the NPs achieved a therapeutic temperature range of 38–39 °C. A specific loss power (SLP) of 27.90 W/g was recorded at a concentration of 1 mg/mL under an AC field strength of 26.67 kA/m. In addition to their hyperthermia potential, the NPs demonstrated promising electrochemical performance, with an energy density of 4.88  Wh/kg and a power density of 419 W/kg, confirming their applicability in supercapacitor devices. Cytocompatibility studies using NRK-52E cell lines at varying concentrations (10–50 mg/mL) confirmed the biocompatibility of the NPs. Overall, this work successfully demonstrates a green-synthesized nanomaterial with multifunctional potential for biomedical and energy-related applications.

## Introduction

Spinel ferrite-based nanostructures (SFNs) have gained considerable interest due to their versatile physicochemical properties, which make them suitable for a wide range of applications, including catalysis, environmental remediation, biosensing, energy storage, and magnetic hyperthermia^1–4^. Their inherent magnetic behavior, stability, and structural tunability offer significant advantages^5^. However, conventional SFNs often face challenges such as cytotoxicity, low surface area, poor aqueous dispersibility, and limited magnetic and electrical performance^6^. To overcome these limitations, metal ion doping has emerged as an effective strategy. Incorporating dopants such as Mn, Ti, Ni, or Co into the spinel lattice enhances conductivity, magnetism, redox activity, and overall stability^7–9^. This approach not only improves material performance but also broadens their applicability. Among various applications, SFNs show particular promise in magnetic hyperthermia for cancer therapy and in supercapacitors for energy storage, owing to their excellent magnetic response and charge storage capabilities. Thus, doped SFNs present a compelling route toward the development of multifunctional nanomaterials for dual biomedical and energy applications^10^. SNFs show other applications like catalysis, chemical sensing, electromagnetic interference shielding, and environmental remediation^7–11^.

Magnetic hyperthermia treatment (MHT) is an emerging and promising therapeutic technique that employs spinel ferrite-based magnetic nanoparticles (SFNs-MNPs) to selectively target and destroy cancer cells. This method involves the application of an alternating current (AC) magnetic field to induce localized heating in the tumor region. When exposed to such a field, magnetic nanoparticles can generate heat and raise the temperature of cancerous tissues to the therapeutic range of approximately 43–44 °C, which is sufficient to induce apoptosis in malignant cells while sparing surrounding healthy tissues from thermal damage^11–14^. The heating efficiency of magnetic nanoparticles in MHT is quantitatively evaluated by their specific loss power (SLP), also known as the specific absorption rate (SAR), which represents the amount of heat generated per unit mass of nanoparticles under specific field conditions^15^. The key mechanism behind this heating is the ability of magnetic nanoparticles to convert electromagnetic energy into thermal energy through Néel and Brownian relaxation processes. Néel relaxation arises from the reorientation of magnetic moments within the nanoparticles, while Brownian relaxation results from the physical rotation of the entire particle in the medium^16^. The localized heating effect induced by SFNs not only facilitates direct thermal ablation of cancer cells but also enhances the efficacy of conventional therapies such as chemotherapy and radiotherapy. By increasing cell membrane permeability and disrupting cellular repair mechanisms, hyperthermia sensitizes tumor tissues, making them more responsive to co-administered therapeutic agents^17^. For successful and efficient MHT, several key parameters must be carefully optimized. These include the magnetic saturation (Ms) of the nanoparticles, which determines the maximum magnetization under an applied field, their biocompatibility to ensure safe application in biological systems, and their size, shape, and surface chemistry to control heating behavior and biodistribution^18^. High-performance SFNs with tailored magnetic and physicochemical properties are, therefore, at the forefront of research in the field of magnetic hyperthermia.

Ti_0.5_Fe_2.5_O_4_ offers tunable magnetic properties through titanium substitution, resulting in enhanced superparamagnetic behavior. Titanium incorporation improves thermal stability and biocompatibility, essential for magnetic hyperthermia applications. The composition also exhibits multifunctionality with promising electrochemical performance for energy storage. Titanium ferrite NPs have demonstrated significant potential in the electronics industry due to their semiconducting properties^19^. These NPs exhibit a unique combination of magnetic and electrical characteristics, making them ideal for various electronic applications. Titanium ferrite NPs also possess high dielectric constants, which improve the performance of capacitors and energy storage systems^20^. Their excellent thermal stability, electrical conductivity, and tuneable magnetic properties make them suitable for use in electromagnetic interference (EMI) shielding, microwave devices, and heating agents, contributing to more efficient, miniaturized, and reliable electronic components^21^. As electrode materials, Titanium ferrite NPs significantly enhance energy storage capacity and accelerate charge–discharge cycles, enabling faster, more reliable energy release. This makes them particularly useful in applications where rapid energy delivery is critical, such as in electronic devices, particularly in supercapacitors. In addition, Magnetic NPs^22–30^ generate thermal energy when exposed to an alternating magnetic field (AMF), widely used in applications like hyperthermia. While AMF is typically used for its ease of generation, recent studies show that high-frequency rotating magnetic fields (RMF) can also effectively produce heating. Experimental setups now demonstrate RMF’s potential in magnetic hyperthermia, offering new possibilities^31^.

This study represents a significant advancement over previous work by addressing the lack of multifunctional nanomaterials capable of performing effectively in both magnetic hyperthermia and supercapacitor applications. While earlier studies have explored Fe₃O₄-based materials for either biomedical or energy storage applications independently, very few have investigated a single, compositionally tuned nanomaterial that can cater to both domains with high efficiency. Our work introduces Ti_0.5_Fe_2.5_O_4_ nanoparticles, synthesized via a simple and scalable polyol method, wherein partial substitution of Fe^3+^ with Ti^4+^ leads to notable improvements in both electrochemical properties (such as specific capacitance and cyclic stability) and magnetic characteristics (such as SAR values and coercivity). The choice of Ti doping is deliberate, as Ti^4+^ modifies the local crystal field and electronic environment, optimizing both conductivity for charge storage and magnetic behavior for heat generation. Moreover, we offer a comprehensive evaluation—including structural, morphological, magnetic, and electrochemical analyses—to establish the correlation between composition, microstructure, and functional performance. This holistic approach not only validates Ti_0.5_Fe_2.5_O_4_ as a strong candidate for multitasking applications but also contributes fundamental insights into the design of dual-functional nanoplatforms, which is currently a sparsely addressed area in the literature. Thus, our study not only presents a novel material composition but also advances the conceptual framework for integrating biomedical and energy storage functions into a unified nanomaterial system. More specifically, this study aims to address a critical gap in the current literature, where limited attention has been given to Ti-substituted Fe_3_O_4_ (specifically Ti_0.5_Fe_2.5_O_4_) nanoparticles that can simultaneously perform efficiently in supercapacitor and magnetic hyperthermia applications. While numerous reports exist on Fe_3_O_4_ and its derivatives for individual applications, multifunctional nanomaterials that combine energy storage with therapeutic heating capabilities remain under-explored.

Here the use of the green route to synthesize Ti_0.5_Fe_2.5_O_4_ NPs with crystallinity, consistent particle size distribution, and improved magnetic characteristics. The fruit shell extract of pomegranate is used in the synthesis of Ti_0.5_Fe_2.5_O_4_ NPs which efficiently chelate and reduce the Fe ions, enabling the formation of NPs. This study effort to address significant issues concerning using Ti_0.5_Fe_2.5_O_4_ NPs in MHT. By optimizing the synthesis options, we intend to generate NPs with better magnetic and thermal attributes, thereby rendering them more suitable for hyperthermia applications. Our work uniquely contributes by demonstrating a simple synthesis route, tailored Ti doping level, and a comprehensive evaluation of both electrochemical and magnetic heating performance. This study’s findings are anticipated to assist in developing more effective and safe magnetic hyperthermia therapies for cancer and the energy sector. This positions Ti_0.5_Fe_2.5_O_4_ as a promising candidate for next-generation dual-purpose nanoplatforms, bridging the gap between energy and biomedical applications.

## Materials and methods

### Materials

Ferric nitrate, Fe(NO_3_)_3_·6H_2_O (99.5%), ammonium hydroxide, NH_4_OH (98%), and Titanium iso-prapoxideTi[OCH(CH_3_)_2_]_4_. All chemicals were of analytical grade and used without further purification.

### Filtrate preparation of pomegranate fruit peels

**Fig. 1:**
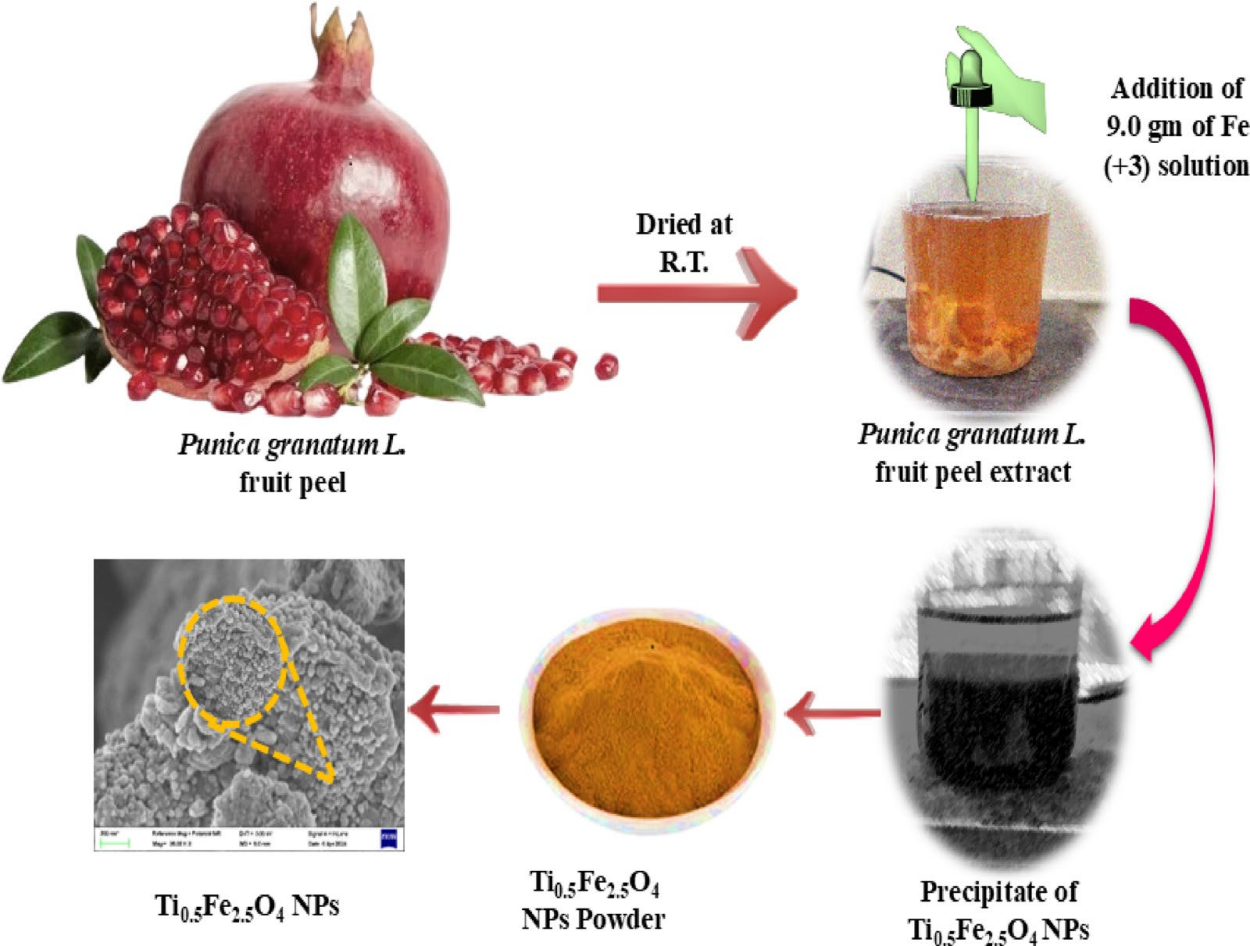
Green synthesis of Ti_0.5_Fe_2.5_O_4_ NPsby ex-situ co-precipitation process.

Pomegranate fruit peels have been taken from local farms in Darikonur (Jat Tahsil), Sangli district, Maharashtra, India. The peels were properly cleaned, air-dried for 9 days at room temperature, and ground to make fine powder. 10 g of fruit peel (powder) were mixed with 100 mL of distilled water (D/W) in a glass beaker and were kept under vigorous stirring for 12 h. After that, residues were separated using Whatman filter paper no.41 and the filtrate was further used as a reducing as well as capping agent (Fig. 1).

### Titano-magnetite nanoparticles (Ti_0.5_Fe_2.5_O_4_ NPs)

Initially, 18.18 mL of titanium oxynitrate [TiO(NO₃)] and 9.0 g of ferric nitrate [Fe(NO_3_)_3_·9H₂O] were transferred to25 mL of D/Wand was ultrasonicated for 20 min. Moreover, 10 mL of pomegranate fruit extract was inserted into the above-sonicated solution under uniform stirring, resulting in a brownish precipitation [1:1 ammonia (pH 9.0 ± 010)]. The precipitate was isolated and dried at 90 °C, followed by washing with ethanol as well as DW, and calcinated at 400 °C for 4 h shown in Fig. 1.

### Characterisation

The crystallite size and crystal structure of Ti_0.5_Fe_2.5_O_4_ NPs were examined using an X-ray diffractometer (XRD, Make-Burker Germany, Model-D8. Advance). Further, different stretching and bending modes of vibrations were examined through a Fourier-transform infrared spectrometer. Fourier-transform infrared (FT-IR) spectroscopy measurements were performed using a Bruker FT-IR spectrometer (Model PN: 1010948106, Germany) to record the stretching frequencies of the functional groups. Spectra were obtained in the range of 400–4000 cm^−1^ with a resolution of 4 cm^−1^. The elemental composition and oxidation state of Ti_0.5_Fe_2.5_O_4_ NPs were analyzed using X-ray photoelectron spectroscopy (XPS), (JEOL Japan and model JPS-9030). Moreover, the topographic and particle size of Ti_0.5_Fe_2.5_O_4_ NPs were studied using a scanning electron microscope (SEM, Carl Zeiss Sigma) and transmission electron microscopy (TEM, Model-JEM-2010, Manufacturer-JEOL, Japan), respectively.

## Results and discussion

### XRD analysis

**Fig. 2:**
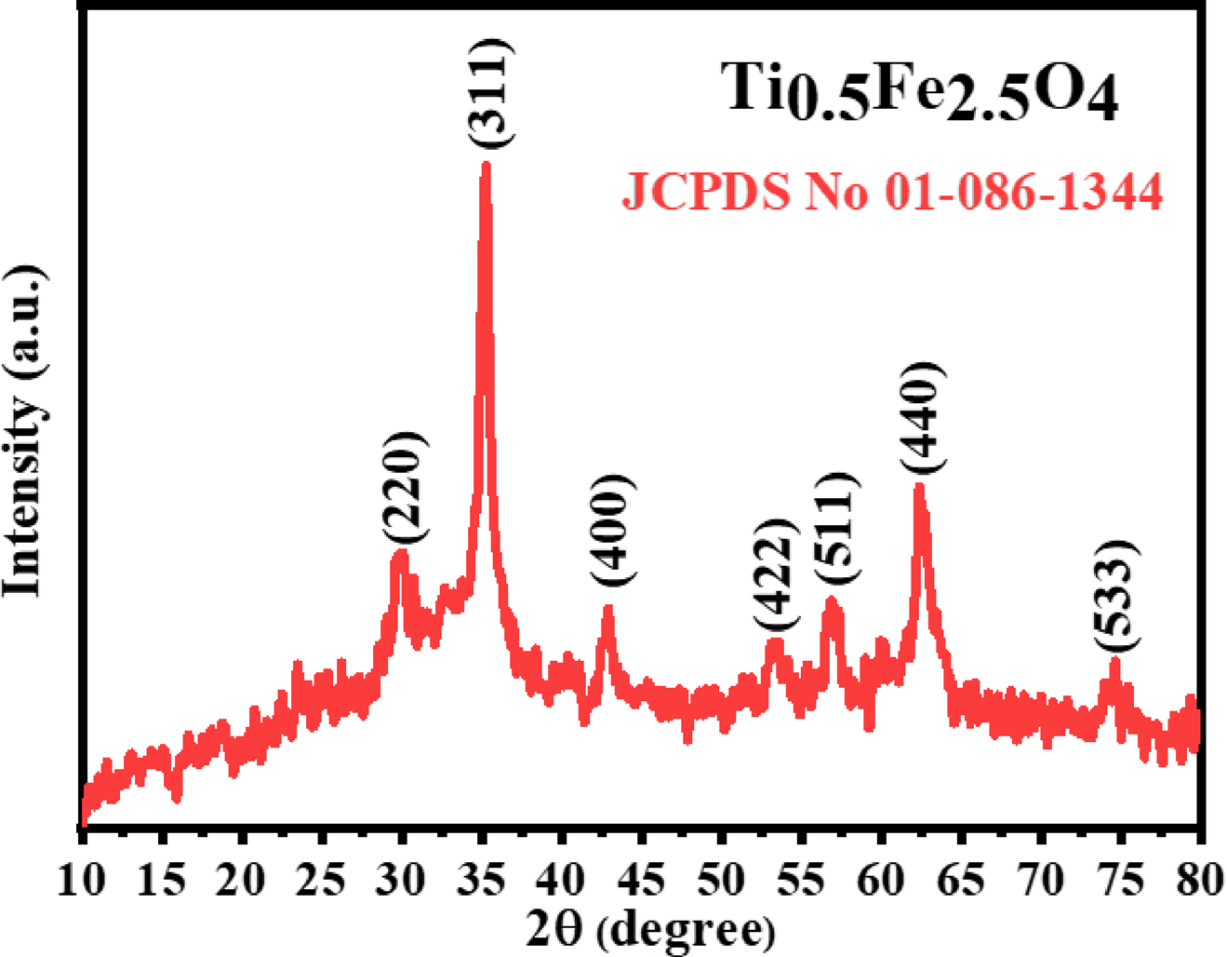
XRD pattern of Ti_0.5_Fe_2.5_O_4_ NPs.

XRD analysis of Ti_0.5_Fe_2.5_O_4_ NPs shows a crystalline spinel structure with distinctive diffraction patterns at 2θ values of 29.90°, 35.13°, 42.96°, 53.34°, 56.92°, 62.32° and 74.73° with corresponding planes (220), (311), (400), (422), (511), (440), and (533) respectively of JCPDS card 01-086-1344, which are indicative of the spinel-ferrite phase^32^. The crystallite size, calculated using the Scherrer equation (Eq. 1), is predicted to be about 16 nm^33^. The XRD pattern illustrates the effective integration of titanium into the iron oxide spinel lattice, resulting in Ti_0.5_Fe_2.5_O_4_ NPs with a cubic crystal structure (Fig. 2). This nanoscale crystallinity is essential to the material’s prospective uses in magnetic and catalytic sectors.

1 $${\text{D}} = \frac{{{\text{n}}\lambda }}{\beta \cos \theta }$$

where 0.9 is the Scherrer’s constant (k), λ is the X-ray wavelength corresponding to Cu Kα, β denotes the fullwidth at half-maximum (FWHM) of the peak and θ is the Bragg angle.

### XPS analysis

**Fig. 3:**
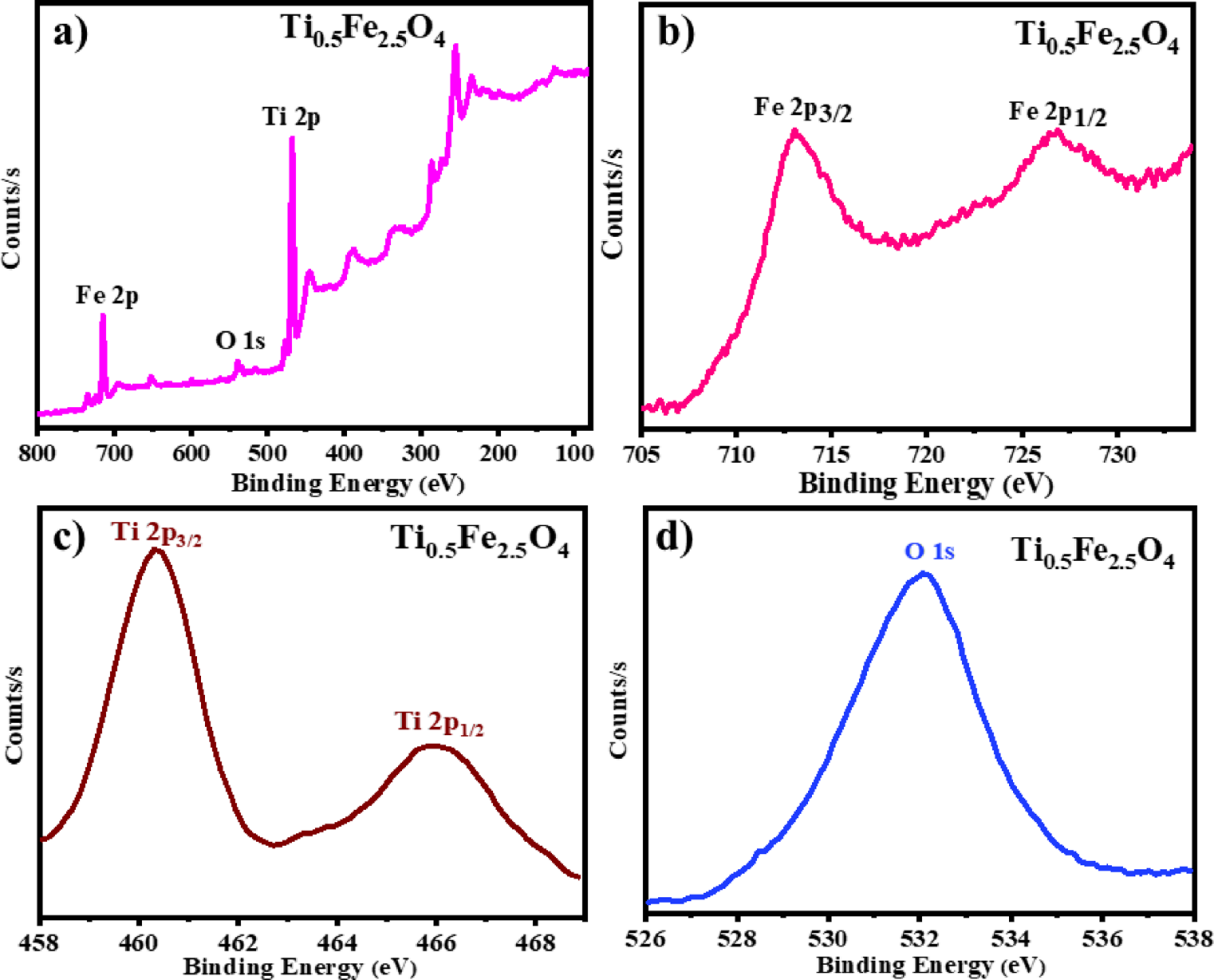
XPS spectra of Ti_0.5_Fe_2.5_O_4_ NPs (**a**) Survey (**b**) Fe element (**c**) Ti element (**d**) O element.

XPS study of Ti_0.5_Fe_2.5_O_4_ NPs reveals its chemical state and binding energies of different elements present in it. The Fe 2p spectra exhibit two distinct peaks at 713.46 eV and 726.74 eV, corresponding to Fe 2p_3/2_ and Fe 2p_1/2_, accordingly, confirming the occurrence of the two forms Fe^2^⁺ and Fe^3^⁺ in the lattice^34^. The Ti 2p region exhibits peaks at 460.54 eV (Ti 2p_3/2_) and 465.81 eV (Ti 2p_1/2_), demonstrating Ti^4^⁺ in the framework^35,36^. The O 1 s spectral has a peak position at 531.73 eV, related to oxygen in the lattice (O^2−^)^37^. The slight shift in the binding energy of Ti_0.5_Fe_2.5_O_4_ NPs confirms the successful incorporation of titanium into the iron oxide spinel lattice, enhancing its complex electronic structure, which is vital for its magnetic and catalytic properties (Fig. 3).

### FT-IR study

**Fig. 4:**
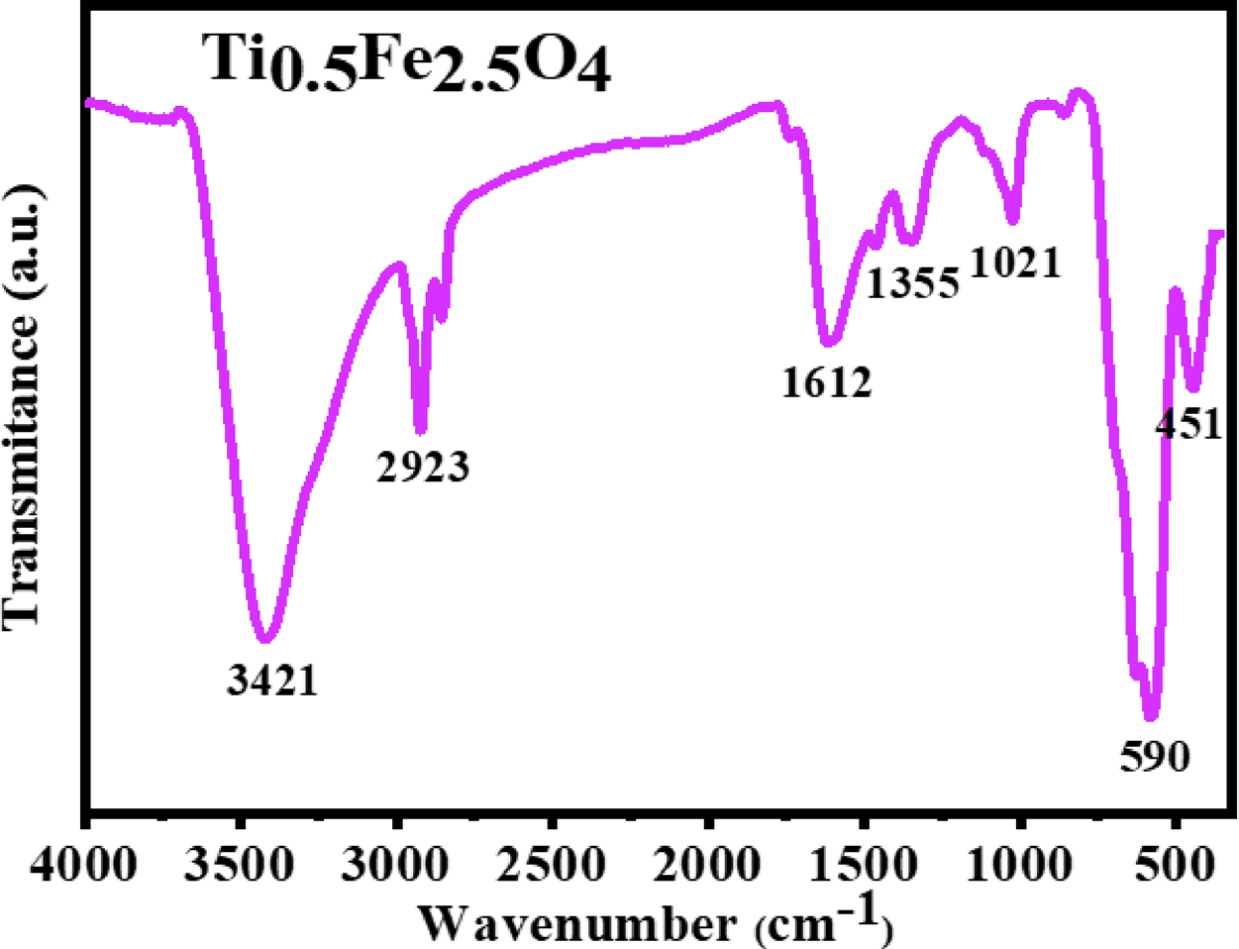
FT-IR spectra of Ti_0.5_Fe_2.5_O_4_ NPs.

The FT-IR spectra of Ti_0.5_Fe_2.5_O_4_ NPs developed using green processes showed peculiar absorption bands associated with the spinel structure. The bands at 451 cm^−1^ and 590 cm^−1^, can be attributed to the Fe–O and Ti–O vibrations of stretching, respectively (Fig. 4)^38^. The 1021 cm^−1^ peak corresponds to C-O stretching, possibly from residual organic matter in the peel extract. The 1612 cm^−1^ peak is due to H–O–H bending vibrations of adsorbed water, while the 1355 cm^−1^ peak suggests C=O or C–O stretching, possibly from carboxyl or carbonyl groups introduced by the green synthesis. The 2923 cm^−1^ peak represents C-H stretching, likely from organic residues or biomolecules from the pomegranate peel extract. Furthermore, the broad peak at 3421 cm^−1^ corresponds to O–H stretching, indicating adsorbed water or hydroxyl groups^39^.

### VSM analysis

**Fig. 5:**
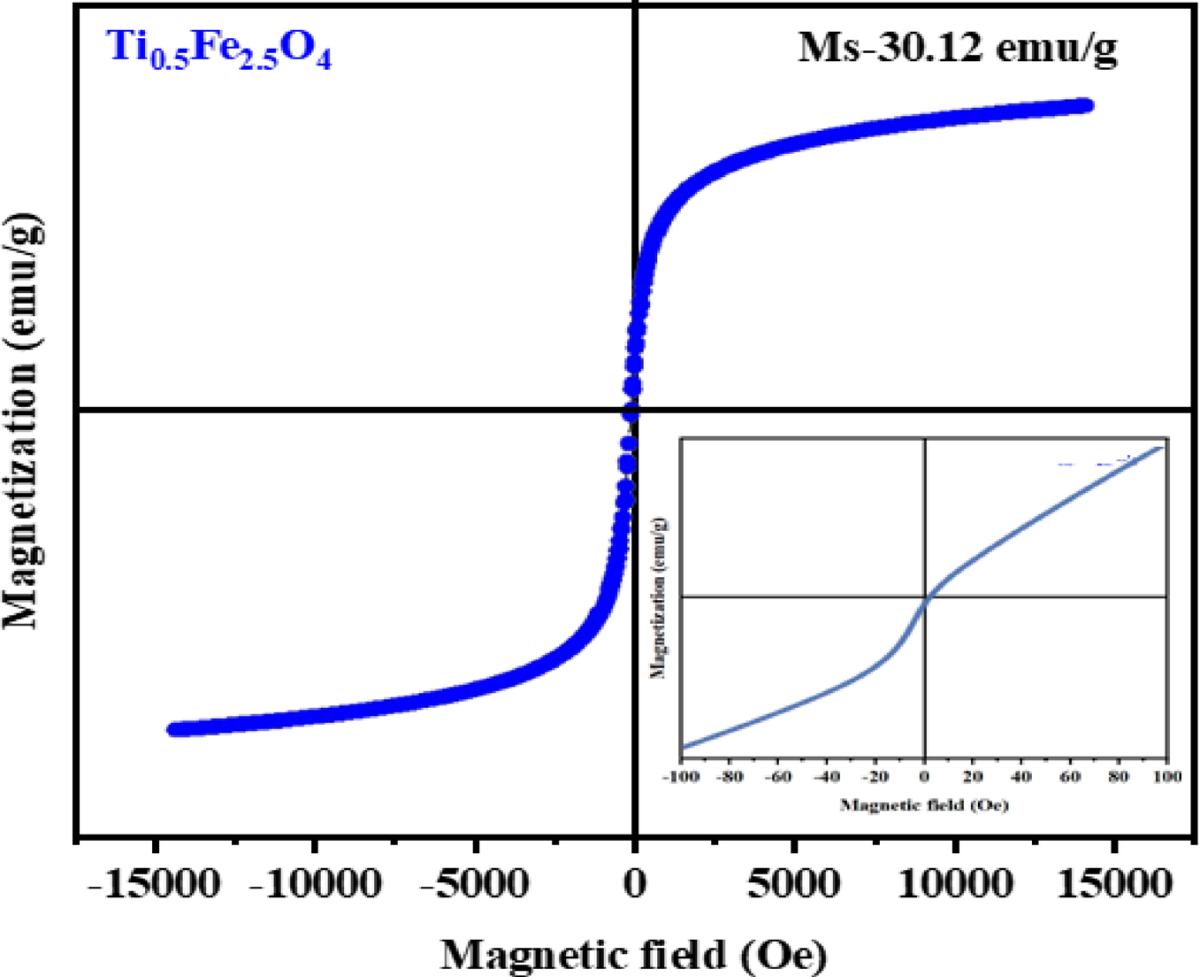
Magnetization of Ti_0.5_Fe_2.5_O_4_ NPs.

The VSM investigation into Ti_0.5_Fe_2.5_O_4_ NPs, reveals its magnetic nature vital to magnetic hyperthermia. The NPs exhibited a saturation magnetization (Ms) of 30.12 emu/g, with zero coercivity (Hc) and remanence (Mr), indicating superparamagnetic behavior at room temperature (Fig. 5). This combination of moderate Ms allows for effective heat production in an alternating magnetic field, making the NPs excellent for hyperthermia therapy. Their magnetic traits instantly enhance therapeutic efficacy by turning magnetic energy into heat, especially driven by Néel and Brownian relaxation phenomena^40,41^.

### Zeta potential

**Fig. 6:**
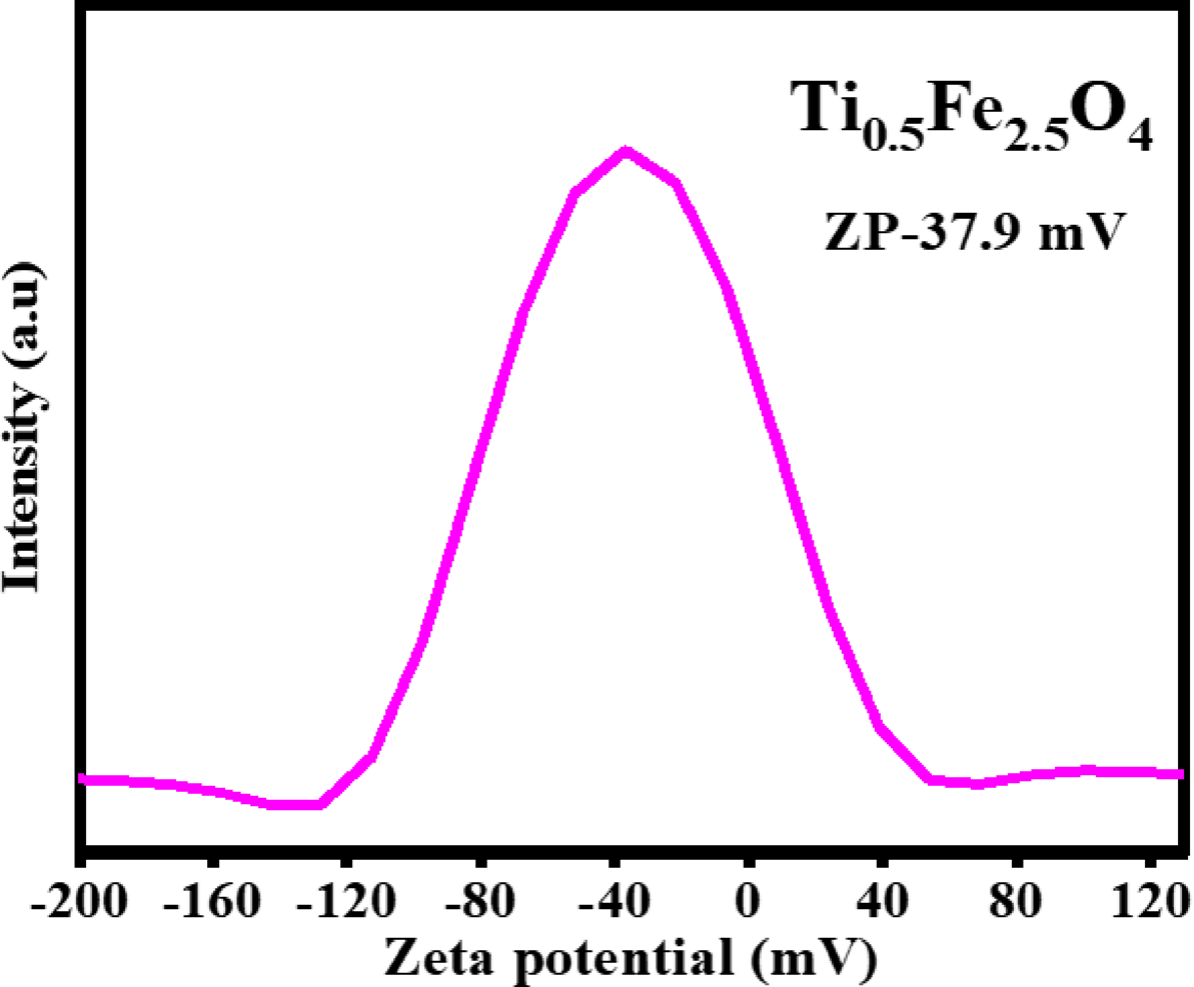
Zeta potential of Ti_0.5_Fe_2.5_O_4_ NPs.

The Zeta potential measurement of Ti_0.5_Fe_2.5_O_4_ NPs reveals a surface charge, indicating colloidal stability and interaction potential in aqueous solutions. Ti_0.5_Fe_2.5_O_4_ NPs exhibit a zeta potential of around − 37.9 mV, indicating a somewhat stable dispersion due to electrostatic repulsion between particles (Fig. 6). Ti_0.5_Fe_2.5_O_4_ NPs possess a high zeta potential, indicating their potential surface charge for biological applications^42^.

### Raman analysis

**Fig. 7:**
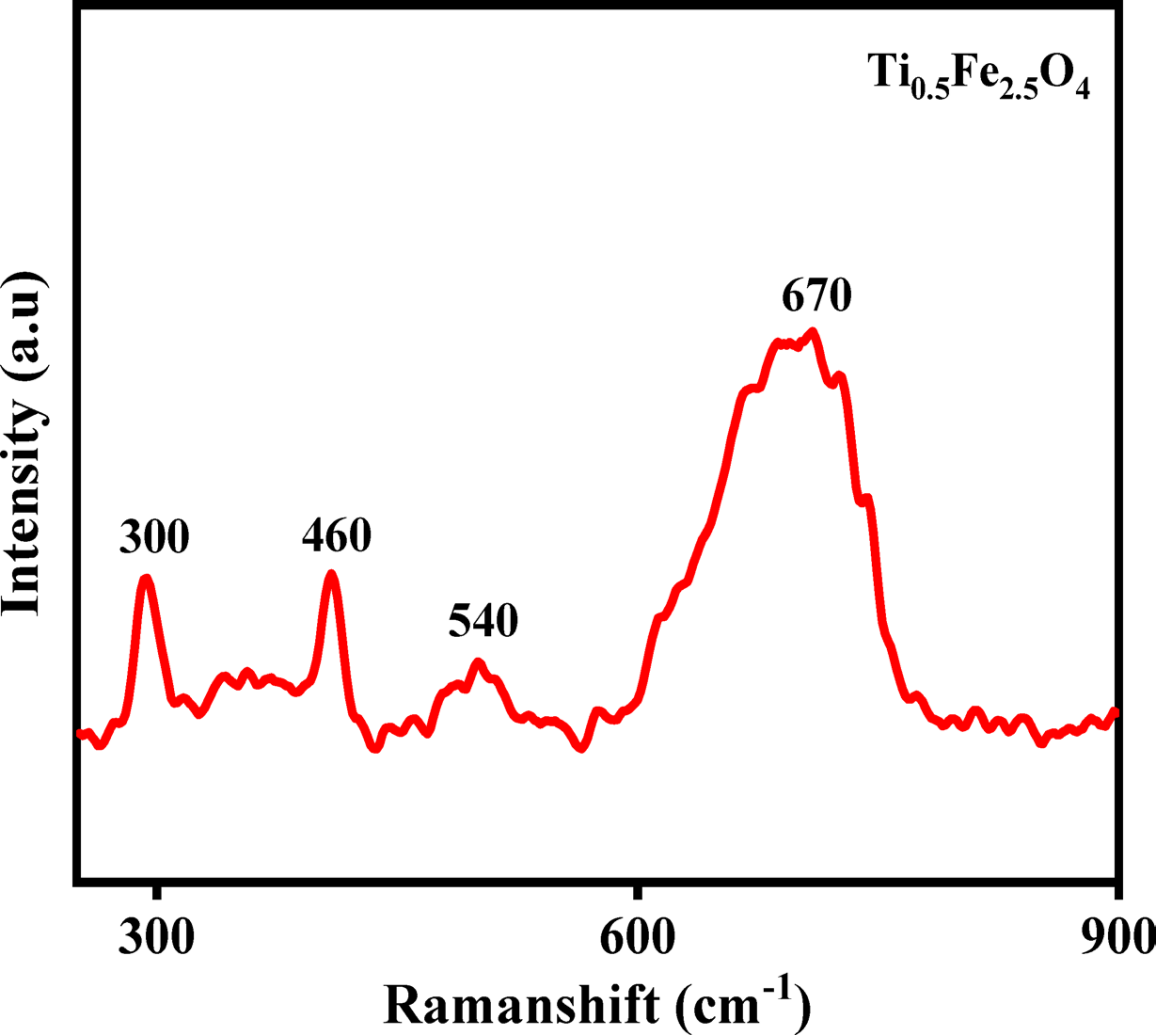
Raman spectra of Ti_0.5_Fe_2.5_O_4_ NPs.

Based on Raman analysis, the Ti_0.5_Fe_2.5_O_4_ sample exhibits characteristic vibrational modes corresponding to a cubic spinel structure (space group Fd$\overline{3}$m). The prominent Raman peaks observed near ~ 670 cm^−1^ (A_1_g), ~ 540 cm^−1^ (T_2_g(3)), ~ 460 cm^−1^ (Eg), and ~ 300 cm^−1^ (T_2_g(2)) confirm the presence of a well-ordered spinel ferrite lattice. The substitution of Ti^4+^ ions at octahedral sites induces slight shifts in these peak positions, attributed to local structural distortions and modifications in the metal–oxygen bond environment (Fig. 7).

### FE-SEM study

**Fig. 8:**
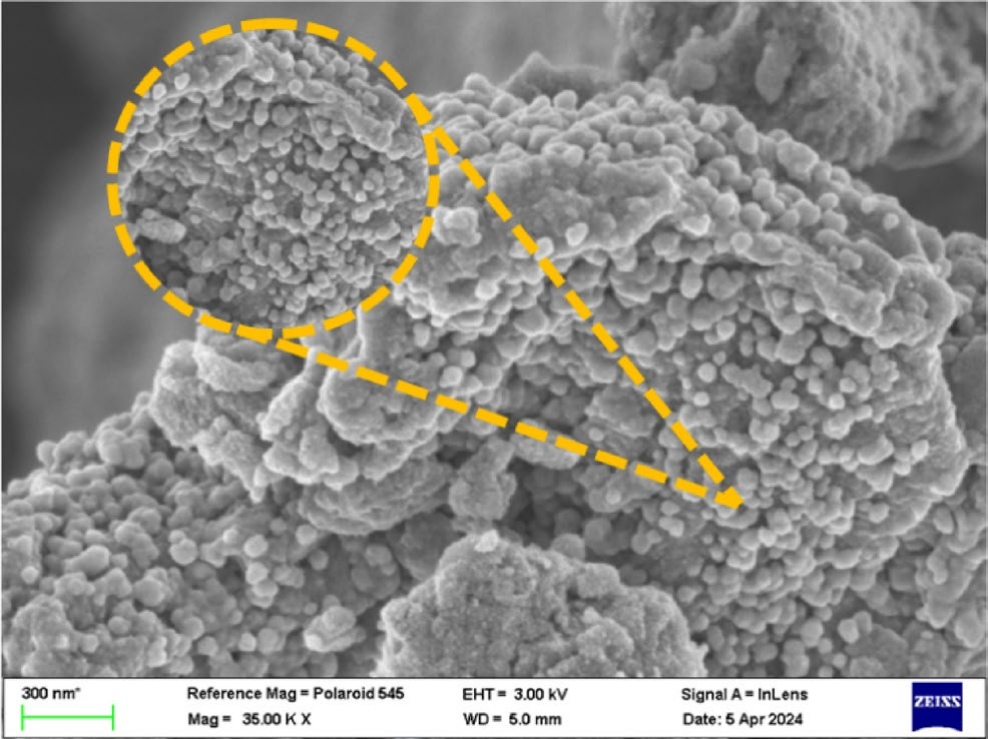
FE-SEM of Ti_0.5_Fe_2.5_O_4_ NPs.

FE-SEM study of Ti_0.5_Fe_2.5_O_4_ NPs reveals uniformly distributed nearly sphere-like NPs via a typical size of 15–20 nm. These particles offer a smooth surface (roughness) and low agglomeration, showing a high degree of consistency and control throughout the synthesis process (Fig. 8). This homogeneous distribution of particles is crucial for maintaining comparable magnetic and catalytic abilities across the specimen, rendering these small particles appropriate for employment in magnetic hyperthermia and other advanced nanotechnologies^43,44^.

### TEM with elemental mapping study

**Fig. 9:**
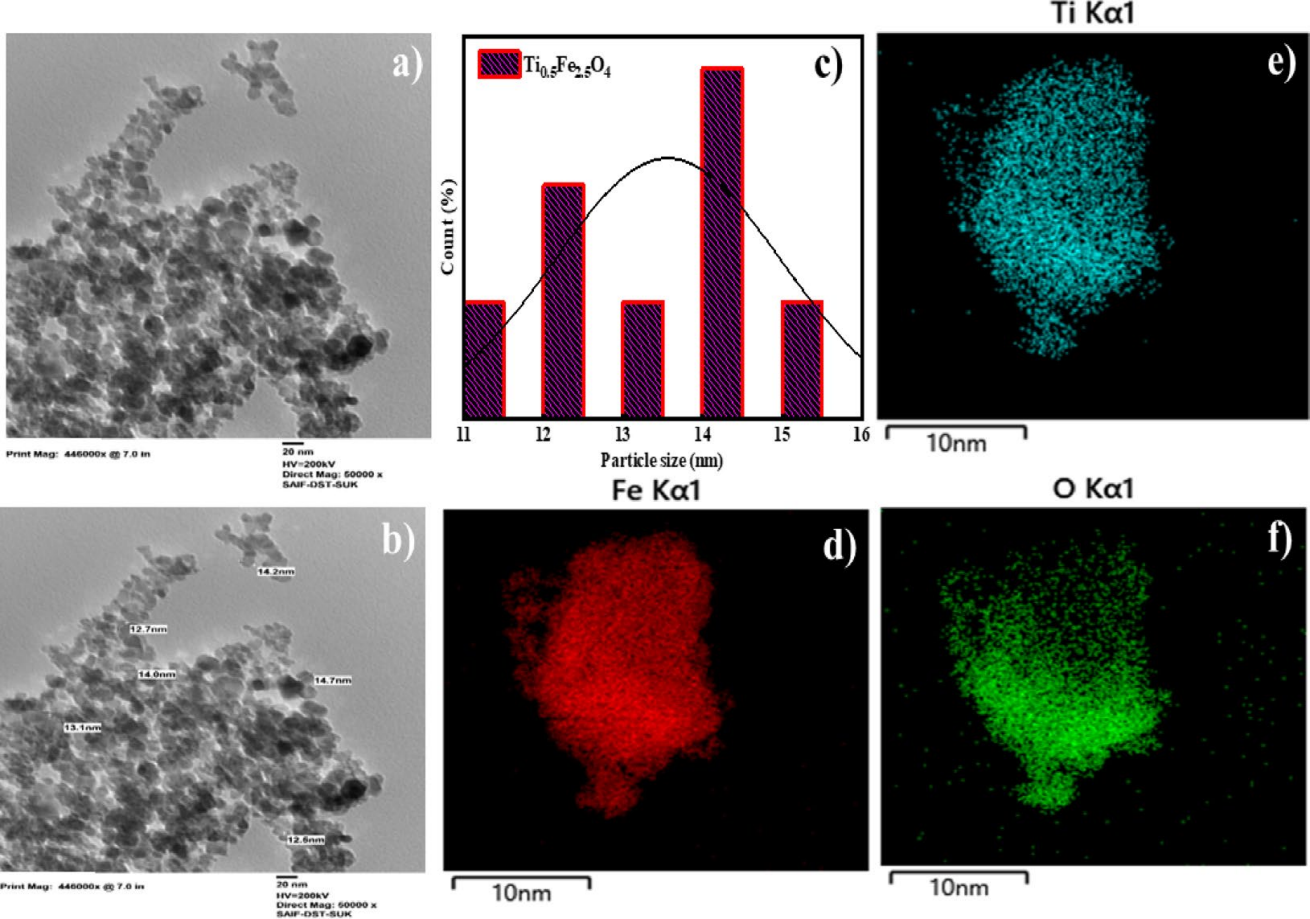
TEM of Ti_0.5_Fe_2.5_O_4_ NPs (**a**, **b**) TEM image, (**c**) APS, and (**d**–**f**) EDS mapping.

Figure 9 indicates the form, size, and crystallinity of Ti_0.5_Fe_2.5_O_4_ NPs. Figure 9c illustrates that nanocrystals are primarily monodispersed, with a particle sizer ranging between 8 and to 18 nm, which improves heat generation efficiency through Néel and Brownian relaxation mechanisms. The EDS assessment reveals an efficient integration of Ti into the spinel lattice, with a uniform element arrangement. Figure 9d–f show the mapping of Ti, Fe, and O distinct distribution, suggesting the predicted stoichiometry and good purity, which validate the nanomaterials’ structural integrity for magnetic and catalytic uses.

### Induction heating system

**Fig. 10:**
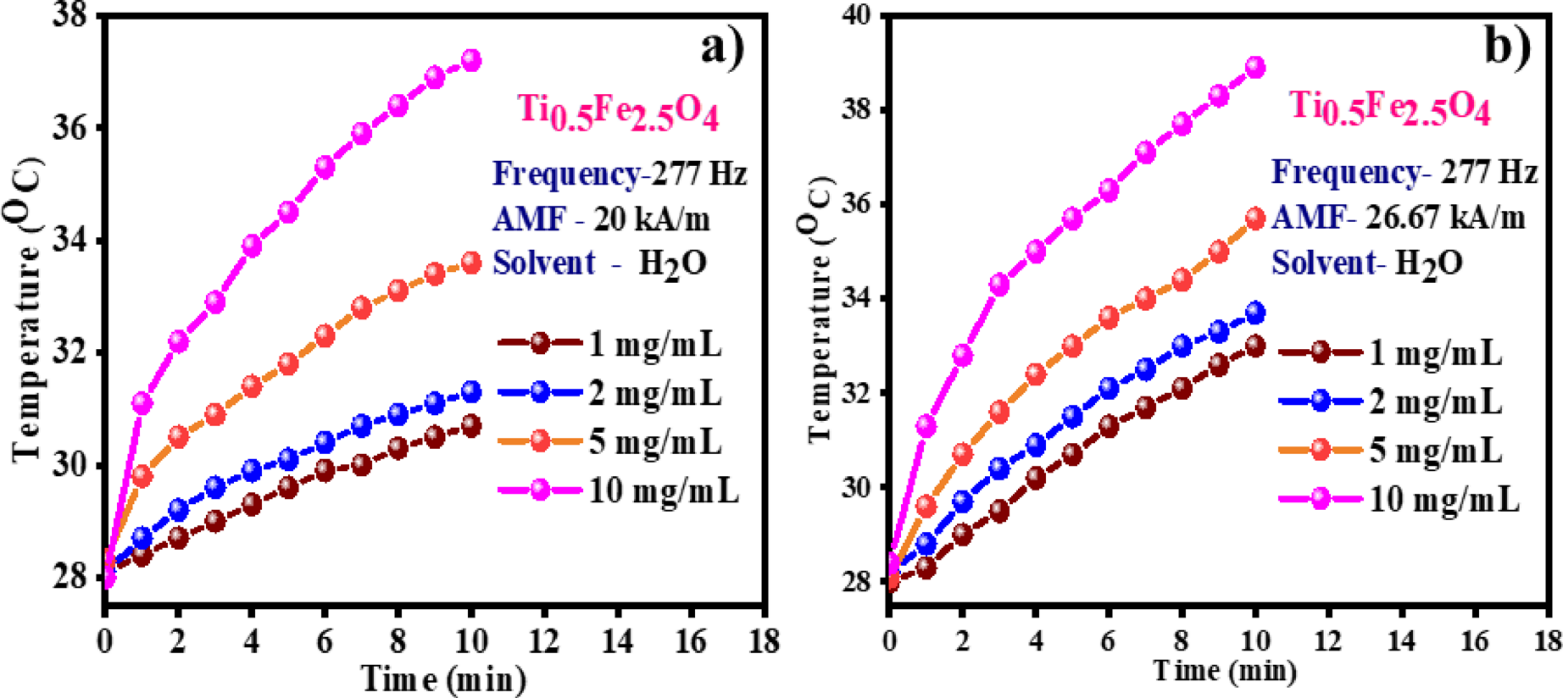
Hyperthermia performance of Ti_0.5_Fe_2.5_O_4_ NPs at a magnetic field of (**a**) 20 kA/m, and (**b**) 26.67 kA/m.

In the present work, heat generated by Ti_0.5_Fe_2.5_O_4_ NPs in a provided alternating magnetic field is employed to quantify hyperthermia performance to kill cancerous cells. Initially, the heating capacity of Ti_0.5_Fe_2.5_O_4_ NPs was noted by varying its concentrations from 1 to 10 mg/mL concerning deionized water and a magnetic field of 20 kA/m as shown in Fig. 10a. The heating profiles for Ti_0.5_Fe_2.5_O_4_ NPs were recorded using Nova Star 5 kW RF Power Supply, induction coils, and a chiller at a frequency of 277 kHz. At first for 1 mg/mL concentration of Ti_0.5_Fe_2.5_O_4_ NPs, the temperature reached up to 30 °C. When the concentration was increased to 2 mg/mL, the heating ability of Ti_0.5_Fe_2.5_O_4_ NPs also enhanced to 32 °C. This heating proficiency even increased for 5 mg/mL (34 °C) and 10 mg/mL (39 °C) at an amagnetic field of 20 kA m^−1^.

Samples were heated for 10 min with the desired current (300–400 A). For the conducted experiments, the magnetic field was calculated from the relationship of Eq. (2).

2 $${\text{H}} = \frac{{1.25{\text{ ni}}}}{{\text{L}}}$$

where n, i and L denote the number of turns, applied current, and diameter of turns in centimeters respectively. Calculated values of the magnetic field (H) at 300 A to 400 A were 251.4 to 335.2 Oe (equivalent to 20 to 26.67 kA/m) respectively. Temperature rise was measured using an alcohol thermometer and an optical fiber probe with an accuracy of 0.1°C^45^.

Similar studies were carried out at an increased magnetic field of 26.67 kA/m to further check the effect of the applied magnetic field on the heating capacity of Ti_0.5_Fe_2.5_O_4_ NPs as illustrated in Fig. 10b. For 1, 2, 5, and 10 mg/mL concentrations of Ti_0.5_Fe_2.5_O_4_ NPs hyperthermia temperatures of 31, 34.2, 33.3, and 39 °C, respectively were observed. So, from the following results, it was observed that with a concentration of Ti_0.5_Fe_2.5_O_4_ NPs applied magnetic field plays a crucial role in reaching close to hyperthermia temperature. In addition, heating efficacy was measured in the form of specific loss power (SLP) values determined employing the initial slope of the heating profile (Eq. 3)^46^.

3 $${\text{SLP}} = {\text{C}}\left( {\frac{{{\text{Ms}}}}{{{\text{Mm}}}}} \right)\frac{{{\text{dT}}}}{{{\text{dt}}}}$$

where C represents the heating capacity, Ms is the mass of the solution, Mm is the mass of NPs, and dT is the differential temperature increase in time dt.

**Fig. 11:**
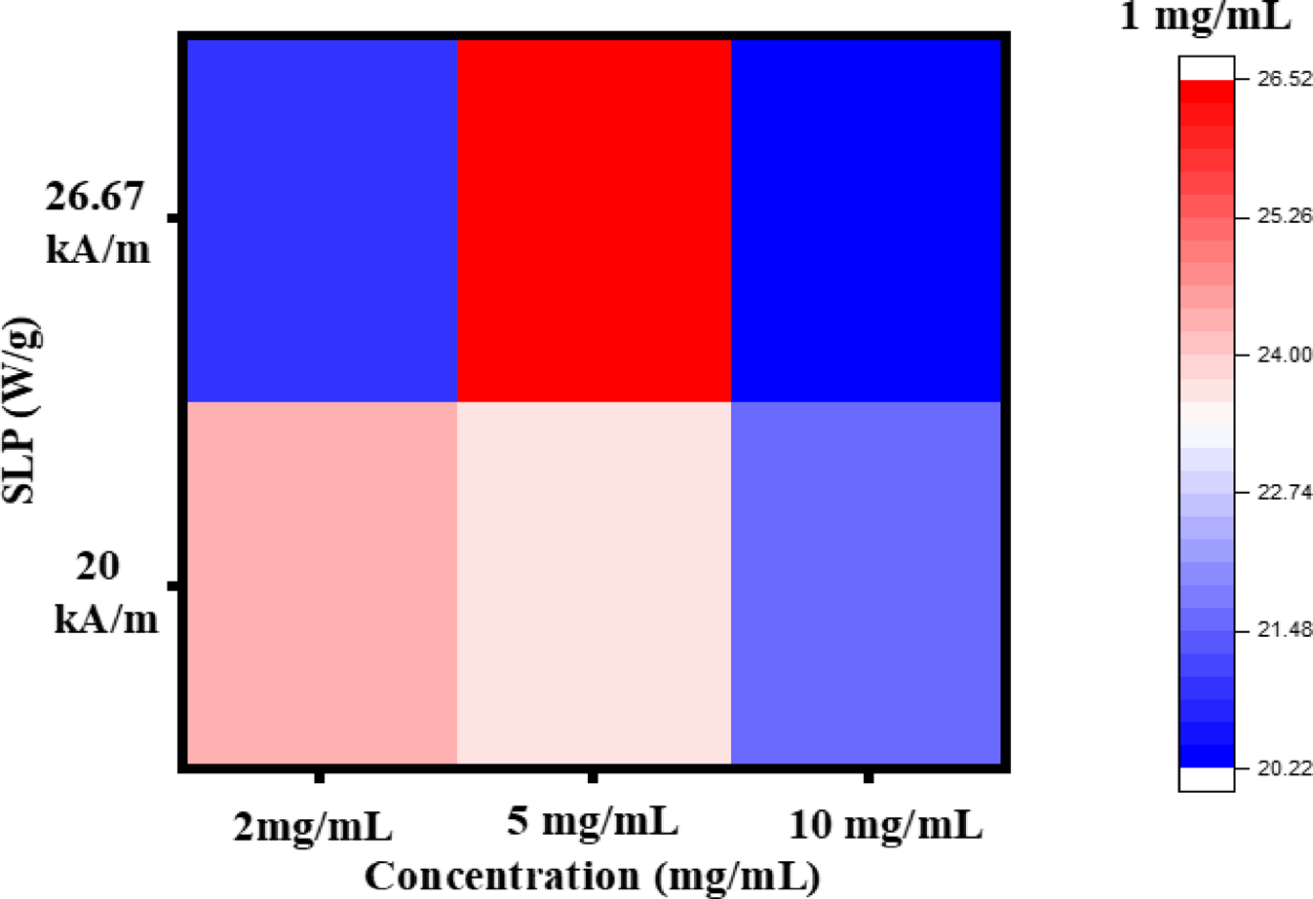
Specific loss power (SLP), of Ti_0.5_Fe_2.5_O_4_ NPs.

The specific loss power (SLP) values of Ti_0.5_Fe_2.5_O_4_ NPs were evaluated at different concentrations (1, 2, 5, and 10 mg/mL) under an applied magnetic field of 20 kA/m. The SLP values recorded were 20.93 W/g, 24.41 W/g, 23.72 W/g, and 21.62 W/g, respectively, indicating a slight variation in heating efficiency across concentrations. When the magnetic field was increased to 26.67 kA/m, the SLP values correspondingly rose, with measurements of 27.90 W/g for 1 mg/mL, 20.93 W/g for 2 mg/mL, 26.51 W/g for 5 mg/mL, and 20.23 W/g for 10 mg/mL, as shown in Fig. 11. This trend illustrates that higher magnetic fields enhance the thermal output of the NPs, though the effect varies slightly with concentration. These findings highlight the promising heating capabilities of Ti_0.5_Fe_2.5_O_4_ NPs, particularly for applications in hyperthermia-based medical treatments, where precise heating is crucial for effective therapy. This behavior suggests that these NPs can be fine-tuned for optimized thermal efficiency at specific concentrations and magnetic field strengths, enhancing their versatility for clinical applications. Moreover, Intrinsic Loss Power (ILP) is a standardized metric used to evaluate and compare the heating efficiency of magnetic NMs in MHT applications. ILP values were calculated using Eq. (4)^47^.

4 $${\text{ILP}} = \frac{{{\text{SLP}}}}{{{\text{f}}.{ }\left( {{\text{Ho}}} \right)2}}$$

where SLP is the specific loss power (W/g), f is the frequency (kHz), and Ho is the amplitude of the applied magnetic field (kA/m or Oe).ILP of 1, 2, 5, and 10 mg/ml at 20 and 26.67 kA/m such as 0.00018871, 0.000220087, 0.000213866, 0.000194931, and 0.000141287, 0.00010599, 0.000134248, 0.000102446 respectively nHm^2^ kg^−1^. In situations of MHT, high ILP values are crucial because they indicate more effective heat generation, implying that treatments can be delivered with less exposure time and adverse effects. Table 1 indicates the literature data compared with our nanoparticles in the form of frequency, hyperthermia performance, synthesis route, and SLP values.

**Table 1:** Comparative study of Ti_0.5_Fe_2.5_O_4_ NPs with other metal oxides.

Sr. No	Materials	Synthesisprotocol	Frequency (kHz)	SAR values (W/g)	Temp. (°C)	References
1	Fe_3_O_4_	Co-precipitation method	265.0	38.4	~ 42	^48^
2	Fe_3_O_4_	Carbothermal reduction method	245.6	43.5	~ 43	^49^
3	ε-Fe_2_O_3_	Sol–gel method	542.0	35.0	~ 38–39	^50^
4	α-Fe_2_O_3_	Hydrothermal route	332.8	03.5	~ 32	^51^
5	Fe_3_O_4_	Solvothermal	120.0	18.5	~ 42–45	^52^
6	Fe_3_O_4_	Solvothermal method	120.0	18.5	~ 42–44	^52^
7	Fe_3_O_4_	–	200.0	70.6	~ 10–15	^53^
8	Fe_3_O_4_	Biosynthesis	290.0	164	~ 40–46	^54^
9	Fe_3_O_4_	Biosynthesis	290.0	286	~ 40–46	^54^
11	Fe_3_O_4_	Co-precipitation method	265	38.4	~ 42	^48^
13	Fe_3_O_4_	Co-precipitation method	175.0	21.4	~ 45	^55^
14	Fe_3_O_4_	Solvothermal	120.0	18.5	~ 50	^52^
15	Fe_3_O_4_	Co-precipitation method	265.0	28.7	~ 60	^56^
16	Ti_0.5_Fe_2.5_O_4_	Green synthesis	277.0	27.9	~ 38–39	This work

### Electrochemical performance

#### Cyclic voltammetry (CV) study

**Fig. 12:**
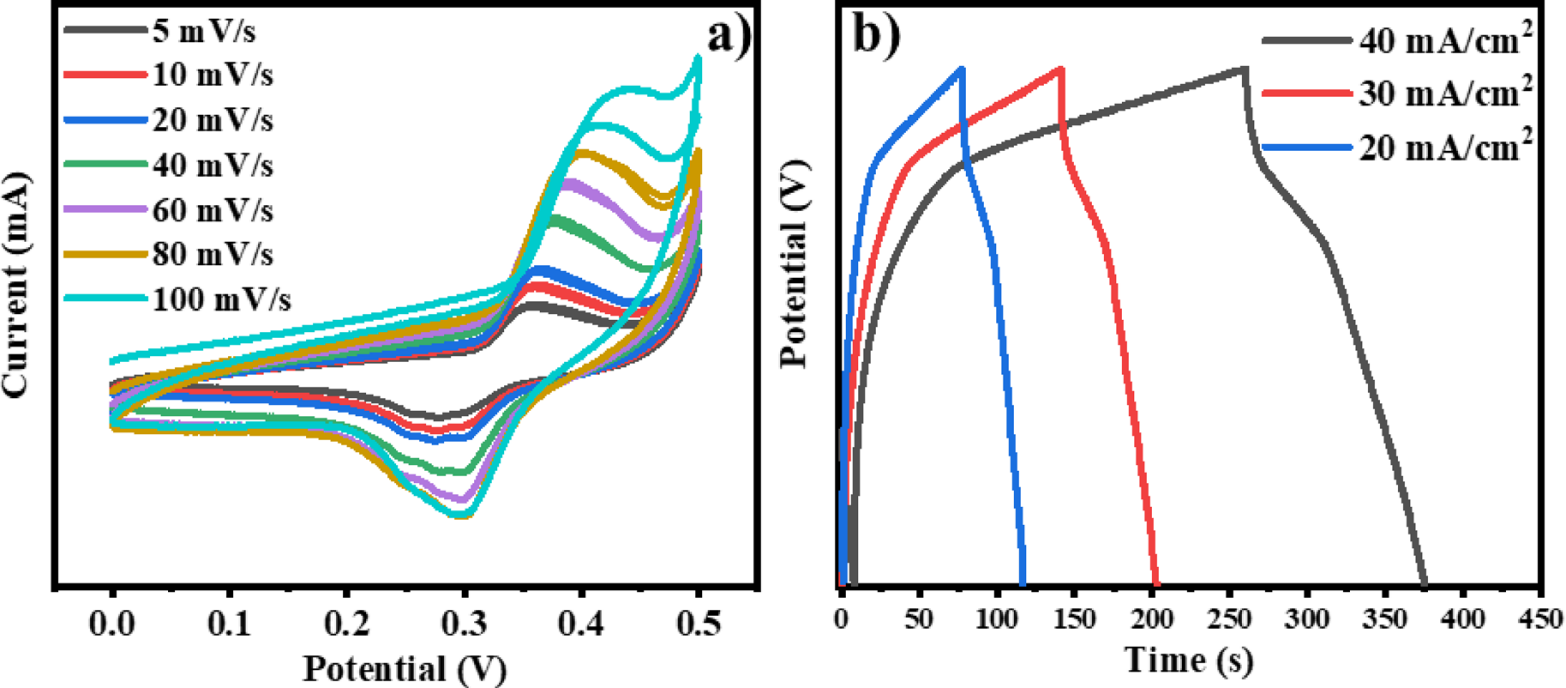
(**a**) CV study of the Ti_0.5_Fe_2.5_O_4_NPsat a different scan rate of 5–100 mV/s, and (**b**) GCD study of the Ti_0.5_Fe_2.5_O_4_ at current densities of 20–40 mA/cm^2^.

CV curves of Ti_0.5_Fe_2.5_O_4_ NPs in a 1 M KOH solution exhibited a non-ideal shape, indicative of Faradic supercapacitor behavior when analyzed at a sweep rate of 5 to 100 mV/s (Fig. 12). This characteristic shape, devoid of prominent redox peaks, suggests a rapid and reversible charge–discharge process, essential for high-performance supercapacitors. The material demonstrated a specific capacitance of 237 F/g under a scan rate of5 mV/s, highlighting its exceptional electrochemical properties (Fig. 12a). However, as the scan rate increased, a slight decrease in capacitance was observed, suggesting the influence of non-Faradaic processes.

Specific capacitance of the materials was calculated using Eqs. (5) and (6) based on CV and GCD data, respectively^49^.

5 $${\text{Specific capacitance}} = \frac{{\text{A}}}{{2{\text{mk}}\,\Delta {\text{V}}}}$$

6 $${\text{Specific capacitance}} = \frac{{{\text{I}} \times \Delta {\text{t}}}}{{\Delta {\text{V}} \times {\text{m}}}}$$

Additionally, the energy density (Ed) (Wh/kg) and power density (Pd) (W/kg) of the Ti_0.5_Fe_2.5_O_4_ NPs were calculated from Eqs. (7) and (8), respectively^35^.

7 $${\text{E}}_{{\text{d}}} = \frac{1}{{2{ } \times { }3.6}}\,\,{\text{Cs}}\left( {\Delta {\text{V}}} \right)^{2}$$

8 $${\text{P}}_{{\text{d}}} = \frac{{{\text{Ed}}}}{{{\text{Td}}}}{36}00$$

#### Galvanostatic charge–discharge (GCD) analysis

The GCD studies were further carried out to evaluate the charge storage capacity of the Ti_0.5_Fe_2.5_O_4_ NPs. GCD profiles of Ti_0.5_Fe_2.5_O_4_ NPs at various current densities, ranging from 20 to 40 mA/cm^2^, displayed a symmetrical triangular shape, further reinforcing their capacitive nature. The electrode exhibited excellent electrochemical stability and reversibility, as evidenced by the linear GCD curves with minimal voltage drop, indicating a highly efficient charge–discharge process (Fig. 12b). The superior specific capacitance of the Ti_0.5_Fe_2.5_O_4_ NPs can be attributed to its unique structural characteristics, including a high specific surface area and low internal resistance. Table 2 compares the capacitance of various materials compared with Ti_0.5_Fe_2.5_O_4_ NPs show higher capacity.

**Table 2:** Comparing electrochemical supercapacitor performance of Ti_0.5_Fe_2.5_O_4_ NPs electrode with previously reported Fe_3_O_4_-based electrode.

Component	Electrolytes	Current density	Capacity retention (%)	Specific capacitances	References
Fe_3_O_4_	1 M KOH	10 A/g	55.0%	241.0 F/g	^57^
Fe_3_O_4_	1 M KOH	0.5 A g1	–	220.1 F/g	^58^
Fe_3_O_4_	3 M KOH	1 mA	–	185.0 F/g	^59^
Fe_3_O_4_	6 M KOH	3 A/g	–	396.0 F/g	^60^
Fe_3_O_4_/TiO_2_@C	1 M Na_2_SO_4_	1 mA/cm^2^	90.7%	304.1 mF/cm^2^	^61^
Ti_0.5_Fe_2.5_O_4_	1 M KOH	20 mA/cm^2^	–	237.0 mF/cm^2^	This work

### Cell viability assay

**Fig. 13:**
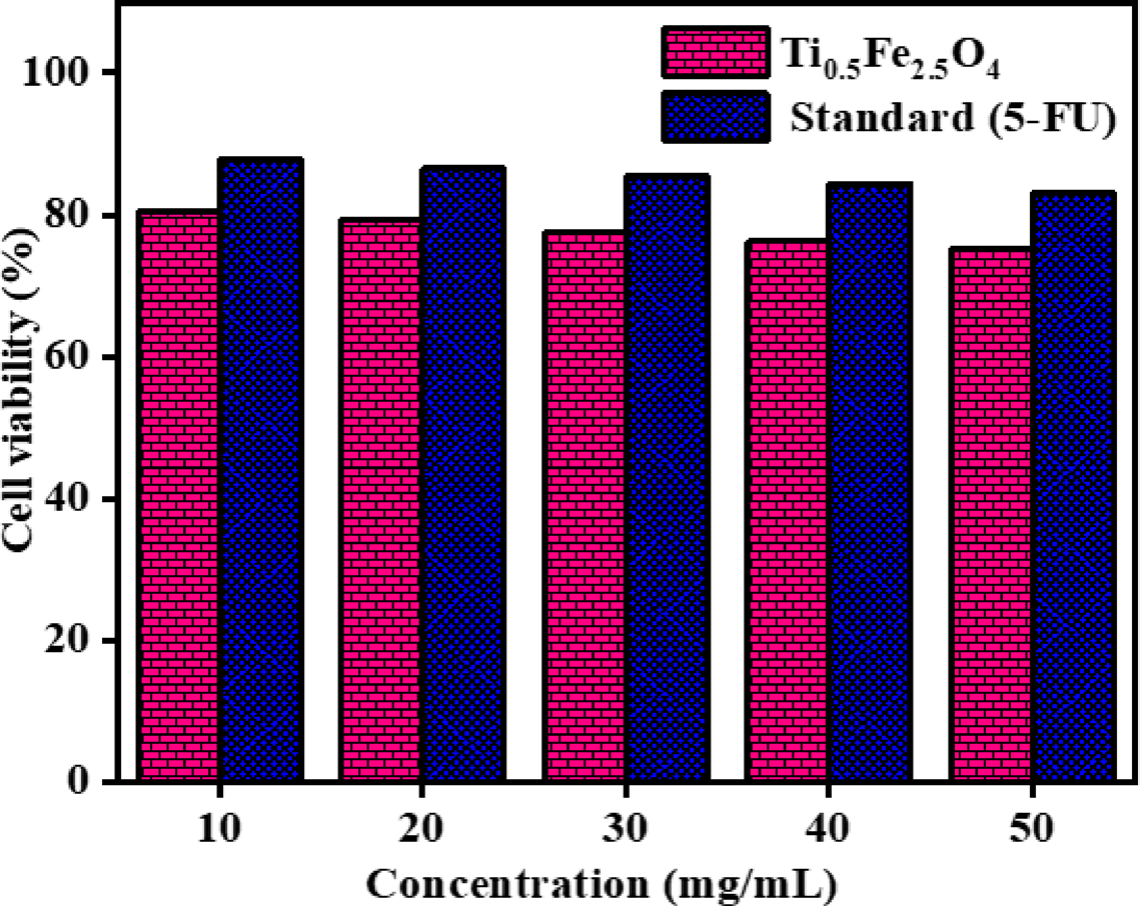
Cell viability of Ti_0.5_Fe_2.5_O_4_ NPs.

Cells were cultured at 1 × 10^4^ cells/mL for 24 h at 37 °C and 5% CO₂. In a 96-well plate, cells were seeded at 10^4^ cells/well in 100 μl of the medium. Sample dosages were 10, 20, 30, 40, and 50 μg/mL. The cell line and control wells were both treated with DMSO (0.2% in PBS). All samples were incubated in triplicate under the same circumstances. After incubation, dispose of the media and add 20 μl of MTT reagent (5 mg/mL PBS). Incubate at 37 °C for 4 h. A microscope was used to inspect wells for the formation of formazan crystals. Viable cells turned yellow MTT into dark formazan (Fig. 13). The viability on the NRK cell line for Ti_0.5_Fe_2.5_O_4_ NPs was approximately 77.33%, up to 24 h. The percentage of cell viability was calculated using Eq. (9).

9 $$\left( \% \right){\text{ Viability}} = \frac{{{\text{Optical density }}\left( {\text{t}} \right)}}{{{\text{Optical density }}\left( {\text{c}} \right){ }}}{ } \times { }100$$

where OD(t) treated is the measurement obtained from cells after 24 h of exposure to 5-FU-loaded Ti_0.5_Fe_2.5_O_4_ NPs. The OD(c) control displays the outcomes of the cells treated with NMs.

In summary, the structural and morphological changes induced by Ti doping in Ti_0.5_Fe_2.5_O_4_ nanoparticles play a crucial role in the observed properties. Ti doping leads to changes in the spinel crystal structure, enhancing the lattice stability and symmetry. This modification improves the charge transfer and electrical conductivity, which is critical for the enhanced electrochemical performance observed in the supercapacitor tests. The introduction of Ti results in more uniformly dispersed nanoparticles with smaller particle sizes and reduced agglomeration. This increased surface area improves electrochemical accessibility for charge storage and also contributes to more efficient magnetic relaxation, enhancing magnetic hyperthermia performance. These structural and morphological optimizations, driven by Ti doping, are directly correlated with the improved energy storage and magnetic heating capabilities observed, making Ti_0.5_Fe_2.5_O_4_ a promising material for multifunctional applications.

The practical implications of this work for industrial and commercial applications include the development of multifunctional nanomaterials for hybrid biomedical and energy devices, such as magnetic hyperthermia-based cancer treatments and energy storage systems like supercapacitors. The scalable, eco-friendly synthesis method and dual functionality of Ti_0.5_Fe_2.5_O_4_ make it a promising candidate for use in cost-effective, multifunctional platforms, potentially advancing both medical therapies and renewable energy solutions.

Moreover, this study provides a clear framework for correlating doping level, nanostructure, and multifunctional performance in spinel oxides, demonstrating how targeted substitution can tune both electrochemical and magnetic properties. By showcasing a scalable, eco-friendly synthesis and comprehensive structure–property analysis, we pave the way for future work to explore other dopant combinations, optimize device integration, and extend multifunctional oxides to applications such as catalysis, sensing, and beyond.

## Conclusion

The present study demonstrates the potential of green-synthesized titano-magnetite (Ti_0.5_Fe_2.5_O_4_) nanoparticles for dual applications in magnetic hyperthermia and energy storage. The nanoparticles exhibited superparamagnetic behavior, efficiently generating heat under an alternating magnetic field and reaching a temperature range of 38–39 °C at 20–26.67 kA/m and 277 kHz. Electrochemical evaluations further revealed their suitability for supercapacitor applications, with an energy density of 4.88 Wh/kg, a power density of 419 W/kg, and a specific loss power (SLP) of 27.90 W/g at a concentration of 1 mg/mL. These results underscore the multifunctional capability of Ti_0.5_Fe_2.5_O_4_ NPs in both biomedical and energy-related domains. Additionally, the NPs demonstrated biocompatibility with NRK 52E normal cells, indicating their potential for safe therapeutic applications.

## Data Availability

The raw/processed data required to reproduce these findings cannot be shared at this time as the data also forms part of an ongoing study and are available from the corresponding author on reasonable request.
